# Pre-Transport Salt Baths Mitigate Physiological Stress and Tissue Damage in Channel Catfish (*Ictalurus punctatus*) Fingerlings: Evidence from Multi-Biomarker Assessment and Histopathology

**DOI:** 10.3390/ani15152249

**Published:** 2025-07-31

**Authors:** Guowei Huang, Haohua Li, Juguang Wang, Tao Liao, Liang Qiu, Guangquan Xiong, Lan Wang, Chan Bai, Yu Zhang

**Affiliations:** 1 School of Life Sciences and Health Engineering, Hubei University of Technology, Wuhan 430068, China; 15572303612@163.com; 2 Key Laboratory of Cold Chain Logistics Technology for Agro-Product, Ministry of Agriculture and Rural Affairs, Institute of Agro-Product Processing and Nuclear Agricultural Technology, Hubei Academy of Agricultural Sciences, Wuhan 430064, China; 3 Agro-Product Processing Research Sub-Center of Hubei Innovation Center of Agricultural Science and Technology, Wuhan 430064, China

**Keywords:** salt bath, juvenile *Ictalurus punctatus*, simulated transport, transport efficiency

## Abstract

Effective transport strategies are critical for the survival and welfare of juvenile *Ictalurus punctatus*, but the effects of pre-transport salt bath treatments remain uncertain. In this study, we systematically evaluated the effects of pre-transport salt bath acclimation at 0‰ (S1), 1‰ (S2), 5‰ (S3), and 9‰ (S4) salinity for 30 min on stress resilience and recovery in fingerlings during 12 h of simulated transport and 24 h of recovery. All fish survived, but total ammonia nitrogen (TAN) increased, and pH decreased in all groups, except S3, which showed significantly lower TAN and higher pH (*p* < 0.05). The S3 and S4 groups showed attenuated increases in serum cortisol and glucose, with S3 exhibiting the fastest return to baseline levels and stable serum sodium and potassium levels. Liver antioxidant enzyme activities in group S3 remained stable, with the lowest malondialdehyde (MDA) accumulation. Integrated biomarker response (IBR) and histological analyses demonstrated that S3 had the lowest systemic stress and tissue damage, whereas S1 and S4 displayed marked cellular disruption. These results indicate that a 5‰ salt bath applied prior to transport may improve water quality, mitigate stress responses, and preserve tissue integrity in juvenile channel catfish. Further studies are needed to confirm these findings in other species and under commercial transport conditions.

## Simple Summary

Transporting juvenile *Ictalurus punctatus* is an important step for fish farmers, but it can cause stress that affects fish health and survival. In this study, we used a simulated transport to test if giving fish a salt bath before moving them could make a difference. We tested different salt bath concentrations and found that a short salt bath with 5‰ salinity before transport helped maintain better water quality, reduced stress, and protected the fish’s tissues. This simple and low-cost method can help fish farmers improve survival rates and reduce losses during live fish transport.

## 1. Introduction

The rapid development of global aquaculture has substantially increased the demand for live fish transportation. According to the Food and Agriculture Organization (FAO), global fisheries and aquaculture production reached 223.2 million tons in 2022, representing a 4.4% increase from 2020 [1]. Among these activities, the transportation of juvenile fish is essential for seed stock distribution and the scaling of aquaculture operations, directly impacting production efficiency and economic returns. Live fish transport overcomes geographical barriers [2], optimizes production cycles [3], and enhances economic value through broader market distribution [4]. Ictalurus punctatus is a commercially important freshwater species, prized for its rapid growth, strong disease resistance, and high meat quality [5,6].

However, live transportation exposes fish to multiple stressors, including fluctuations in water quality, handling, and road conditions [7,8]. These stressors trigger physiological responses that can reduce survival rates, impair growth, and compromise disease resistance, ultimately limiting the sustainability of aquaculture. To monitor and understand these effects, biomarkers, such as cortisol, glucose, and antioxidant enzymes, are widely used as critical indicators of transport-induced physiological disturbances in fish [9,10].

To mitigate transport stress, chemical additives, such as herbal extracts, antibiotics, and antioxidants, have been commonly employed in aquaculture. For instance, herbal extracts like *Lippia alba* can reduce ion loss and improve redox homeostasis, though their effects are dose-dependent and excessive use may elevate plasma cortisol (COR) and induce hepatic oxidative stress [11,12,13,14]. Although antibiotics can promote fish health, their inefficient metabolism and excretion result in environmental contamination and public health risks, leading many countries to restrict or ban their use in aquaculture [15]. Vitamins C and E, as natural antioxidants, have been shown to enhance immunity, growth, and meat quality [16,17]. However, their instability, high cost, and limited effectiveness constrain their widespread application [18]. Thus, while such additives may offer temporary relief from stress, their ecological risks and economic burdens underscore the need for safer and more sustainable alternatives.

Recently, sodium chloride (NaCl) has emerged as a promising and sustainable strategy for alleviating transport-induced stress in fish due to its low cost, ease of administration, and environmental compatibility [19,20]. NaCl helps regulate intracellular and extracellular osmotic pressure, maintains homeostasis, and reduces cell swelling during environmental fluctuations [21]. Its antioxidant properties have been shown to enhance superoxide dismutase (SOD) activity, thereby reducing liver damage caused by reactive oxygen species (ROS) [22]. Moderate salinity has also been shown to enhance growth and osmoregulation in various species, such as juvenile *Trachinotus marginatus* and *Cyprinus carpio*, and to mitigate the loss of water and essential minerals during transport [23,24]. Furthermore, NaCl poses no risk of drug residue accumulation and complies with strict aquaculture regulations [25]. Despite the increasing adoption of salt baths in fish transport, few studies have systematically evaluated their pre-transport effects, particularly in juvenile Ictalurus punctatus. Most previous research has focused on general transport stress or post-transport recovery, with limited attention to the physiological and histological impacts of pre-transport salt bath treatments during and after simulated transport.

To comprehensively assess the efficacy of salt baths, biochemical biomarkers (such as cortisol, glucose, and antioxidant enzymes) and tissue-level histopathology (gills, skin, and intestines) were evaluated.

Given these considerations, this study aims to address the knowledge gap by systematically investigating the effects of pre-transport salt bath acclimation on water quality, stress responses, antioxidant capacity, and tissue integrity in juvenile Ictalurus punctatus during simulated transport. Through an integrated multi-biomarker and histological approach, we seek to elucidate the protective effects of salt baths and provide practical recommendations for optimizing live fish transport and enhancing aquaculture outcomes.

## 2. Materials and Methods

### 2.1. Animals

Juvenile *Ictalurus punctatus* were obtained from Xianning, Hubei Province, China. The fish had an average body weight of 16.22 ± 0.82 g and an average total length of 13.4 ± 0.35 cm. Healthy individuals exhibiting good growth performance were quickly transported to the experimental facility in 1-ton tanks, minimizing oxygenated freshwater. Black plastic sheeting was used to cover the tanks to minimize transport-related stress. Before the experiment, fish were acclimated in a recirculating aquaculture system consisting of cylindrical plastic tanks (1.5 m diameter, 1.3 m height) filled with tap water that had been aerated for two days. Rearing water parameters were maintained as follows: stocking density of 0.3 g/L, temperature at 23.0 ± 0.5 °C, ammonia nitrogen below 0.02 mg/L, dissolved oxygen above 6 mg/L, and pH at 7.12 ± 0.21. During acclimation, fish were exposed to the natural indoor light/dark cycle typical of December in Wuhan, China. The light intensity, measured using a quantum sensor, ranged from 10 to 100 lx. This range was determined based on measurements taken during the present experiment. The health status of the juveniles was monitored daily. Fish were fed commercial feed twice daily (at 9:00 and 16:00) at a rate of 2% of their body weight. Feeding was suspended for 24 h prior to the start of the experiment. All experimental procedures were approved by the Experimental Animal Resource Committee of the Hubei Academy of Agricultural Sciences.

### 2.2. Experimental Design

Salt bath treatments were conducted with a fish-to-water mass ratio of 1:4 (m_fish:m_water = 1:4), following the optimal protocol established by Li Haohua et al. [26]. The selection of a 30 min salt bath duration was based on our preliminary experimental results (see Supplementary Materials for details). Four salt concentrations were applied: S1 = 0‰, S2 = 1‰, S3 = 5‰, and S4 = 9‰, as determined by preliminary screening. Juvenile *Ictalurus punctatus* were randomly selected from the rearing tanks and assigned to four 60 L tanks, each containing 40 L of water and 10 kg of fish (approximately 616 individuals, with an average body weight of 16.22 ± 0.82 g). For each experimental group, the required amount of salt was weighed and gradually added to the designated water volume with continuous stirring until the salt was completely dissolved and no visible precipitate remained. After the salt was fully dissolved, the solution was stirred for an additional 10 min to ensure homogeneity. During the 30 min salt bath, dissolved oxygen (DO) was monitored and maintained at pretreatment levels (DO ≥ 6.0 mg/L) using a portable DO meter.

After the salt bath, juvenile fish of uniform size were randomly selected from each group and transferred to oxygenated, open plastic boxes (21 cm × 13.9 cm × 8.5 cm, with a maximum volume of 2.48 L), following the local standard fish-to-water mass ratio of 1:4 for transportation. Each transport box was filled with 1 L of aerated water and stocked with approximately 15 fish, with continuous aeration to simulate actual transport conditions. During both the salt bath and simulated transport processes, water temperature was maintained at 20 ± 1 °C using an automatic temperature control system and continuously monitored with a digital thermometer to ensure temperature stability. For each salt bath treatment group, five replicate transport boxes (*n* = 5) were set up at 0, 2, and 6 h of simulated transport, and ten replicate boxes were set up at 12 h (five of which were used for subsequent post-transport recovery experiments) for a total of 15 transport boxes per group. A blank control group was maintained under rearing conditions and was not subjected to any salt bath or simulated transport. Each transport box was considered an independent replicate.

### 2.3. Sample Collection and Processing

Each salt bath treatment group included five biological replicates. At each simulated transport time point (0, 2, 6, and 12 h), 15 fish were randomly selected from each box. Blood samples were collected from the caudal vein of five fish per group using 1 mL sterile syringes pretreated with sodium heparin. The samples were centrifuged at 3000 rpm for 10 min using a 3k15 high-speed refrigerated centrifuge (Beijing Huawei Zhongyi Technology Co., Ltd., Beijing, China), and the resulting serum was stored at −80 °C for subsequent physiological and biochemical analyses. Additionally, five fish were randomly selected for liver (1.0 ± 0.1 g) tissue collection for further physiological and biochemical assessment. The remaining fish were dissected on ice to collect skin, gill, and intestinal tissues, which were fixed in 4% paraformaldehyde for hematoxylin–eosin (HE) histological analysis. At each time point, 200 mL water samples were also collected for subsequent water quality analysis.

### 2.4. Water Quality and Survival Rates

The cumulative mortality rate of juvenile *Ictalurus punctatus* was monitored during the simulated transport process (0–12 h) and the subsequent recovery period (0–24 h) to assess the survival status of each group.

Water samples were collected at each designated time point during simulated transportation. Dissolved oxygen (DO) and temperature (°C) were measured using a multi-parameter digital analyzer. The pH was determined with a PHS-25 pH meter, and total ammonia nitrogen was quantified using a portable ammonia nitrogen analyzer.

### 2.5. Serum and Organ Physicochemical Indices

The physicochemical parameters of serum and organs were determined using commercial assay kits (Nanjing Jiancheng Bioengineering Institute, Nanjing, China), following the manufacturer’s instructions. Serum glucose (GLU, A154-1-1, Nanjing Jiancheng Bioengineering Institute, Nanjing, China) was measured using the glucose oxidase method. Serum cortisol (COR, H094-1-2, Nanjing Jiancheng Bioengineering Institute, Nanjing, China) was quantitatively determined using an enzyme-linked immunosorbent assay (ELISA) kit (Nanjing Jiancheng Bioengineering Institute, Nanjing, China). Serum Na^+^ (C002-2-1, Nanjing Jiancheng Bioengineering Institute, Nanjing, China) and K^+^ (C001-2-1) concentrations were measured using a microplate method. Liver superoxide dismutase (SOD, A001-3, Nanjing Jiancheng Bioengineering Institute, Nanjing, China) activity was determined using the WST-1 method. Liver catalase (CAT, A007-1-1, Nanjing Jiancheng Bioengineering Institute, Nanjing, China) activity was measured using the ammonium molybdate method. Liver malondialdehyde (MDA, A003-1, Nanjing Jiancheng Bioengineering Institute, Nanjing, China) content was assessed by the thiobarbituric acid (TBA) method. Liver total antioxidant capacity (T-AOC, A015-2-1, Nanjing Jiancheng Bioengineering Institute, Nanjing, China) was determined using the ABTS method. Liver lactate dehydrogenase (LDH, A020-2-2, Nanjing Jiancheng Bioengineering Institute, Nanjing, China) activity was measured using a microplate method. All assay results were analyzed according to the manufacturer’s instructions.

### 2.6. IBR Indices

The IBR index was calculated according to the method outlined by Beliaeff et al. [27]. First, the mean value (Xi) of a specific biomarker in each group (blank control group, S1, S2, S3, and S4) was determined along with the overall mean (X) and standard deviation (S) for the biomarker across all experimental groups. The average value for each treatment group was then normalized using the formula Y = (Xi − $\bar{X}$ )/S.

The biomarker response to stress was assessed by determining whether it was inhibited. If inhibited, Z = −Y; otherwise, Z = Y. The absolute value of the minimum normalized data for each biomarker in a treatment group is denoted as |X min|. The biomarker score (S) for each treatment group is then calculated as S = Z + |X min|.

The formula for constructing the star diagram is as follows:

(1) $$A_{i}=\frac{S_{i}}{2}\sin\beta \left(S_{i}\cos\beta +S_{i+1}\sin\beta \right)$$

(2) $$\beta =\mathrm{Arc}\tan\left(\frac{S_{i+1}\sin\alpha }{S_{i}-S_{i+1}\cos\alpha }\right)$$

(3) $$IBR=\sum_{i=1}^{n}A_{i}$$

Here, n represents the total number of selected biomarkers, and α is the angle between adjacent radial lines, where α = 2π/n. Ai represents the area connecting the two scores (S), and Si and Si + 1 denote two consecutive clockwise scores (radius coordinates) in the star diagram.

### 2.7. Tissue Sectioning

Fresh skin, gill, and intestinal samples were fixed in 4% paraformaldehyde. After fixation, the tissues were removed from the fixative and trimmed with a scalpel in a fume hood. The trimmed samples along with their corresponding labels were placed in dehydration cassettes and processed through a graded ethanol series using a tissue processor (75% ethanol for 4 h, 85% for 2 h, 90% for 2 h, 95% for 1 h, absolute ethanol I for 30 min, and absolute ethanol II for 30 min). After dehydration, the tissues were cleared sequentially with alcohol–xylene (5–10 min), xylene I (5–10 min), and xylene II (5–10 min). They were then infiltrated with molten paraffin wax at 65 °C in three stages (paraffin I for 1 h, paraffin II for 1 h, and paraffin III for 1 h) and embedded using a paraffin embedding station. The paraffin blocks were sectioned at a thickness of 4 μm using an RM 2016 rotary microtome (Leica Instruments, Shanghai, China). The sections were stained by immersion in hematoxylin solution for 3–5 min, rinsed with tap water, differentiated using a hematoxylin differentiation solution, and rinsed again. Subsequently, the sections were immersed in 85% ethanol for 5 min, 95% ethanol for 5 min, and then in eosin for 5 min. Following final dehydration, the sections were mounted with neutral balsam. Finally, microscopic examination, image acquisition, and qualitative analysis were conducted using a TI-SR inverted fluorescence microscope (Nikon Eclipse, Nikon Corporation, Tokyo, Japan) equipped with a DS-U3 imaging system (Nikon Corporation, Tokyo, Japan) and analyzed with NIS-Elements software (version 6.10.01, Nikon Corporation, Tokyo, Japan).

### 2.8. Statistical Analysis

Experimental results are expressed as mean ± standard deviation (mean ± SD). Statistical analyses were conducted using SPSS 19.0. The Shapiro–Wilk test and Levene’s test were applied to assess normality and homogeneity of variances, respectively. Differences among treatments were analyzed using one-way analysis of variance (ANOVA), followed by Duncan’s post hoc test. *p* < 0.05 was considered statistically significant. Graphs were generated using GraphPad Prism 9 and Microsoft Excel 2020.

## 3. Results

### 3.1. Water Quality and Survival Rates

Continuous monitoring of simulated transportation and post-transport recovery revealed that all juvenile Ictalurus punctatus survived 12 h of simulated transportation and 24 h of post-transport recovery. DO levels were consistently maintained at ≥6.0 mg/L by continuous aeration.

**Figure 1:**
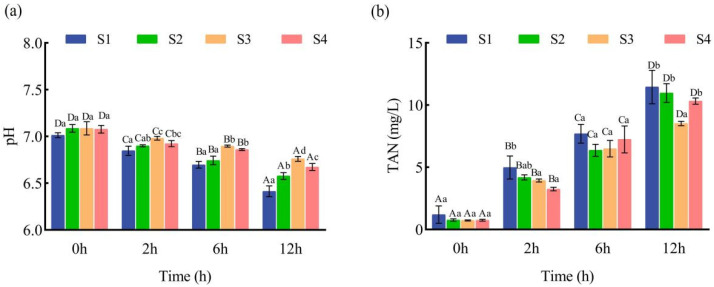
Effect of salt treatment on water quality for juvenile fish transportation. (**a**) pH and (**b**) total ammonia nitrogen (TAN) content (mg/L). Data are presented as mean ± SD (*n* = 5); different capital letters represent significant differences between different sampling times under the same treatment conditions (*p* < 0.05), and different lower-case letters represent significant differences between different concentrations of pre-transportation salt bath treatments under the same sampling time (*p* < 0.05).

As shown in Figure 1, within-group analyses indicated that TAN levels increased significantly, while pH values decreased significantly, with extended transport time in all experimental groups (*p* < 0.05, *n* = 5 per group per time point). After 12 h of simulated transport, the S3 group exhibited the lowest TAN and highest pH among all treatments (*p* < 0.05). Between-group comparisons revealed that TAN levels in the S2, S3, and S4 groups were lower, and pH values were higher than those in the S1 group.

These findings indicate that pre-transport salt bath treatment, especially in the S3 group (5‰ salt bath), effectively maintains water quality by reducing ammonia accumulation and stabilizing pH during transportation. Conversely, the marked increase in TAN and decrease in pH observed in the S1 group (0‰ salt bath) reflect greater metabolic waste buildup and acidification, indicating higher physiological stress in fish under standard transport conditions.

### 3.2. Serum Physiological and Biochemical Indicators

**Figure 2:**
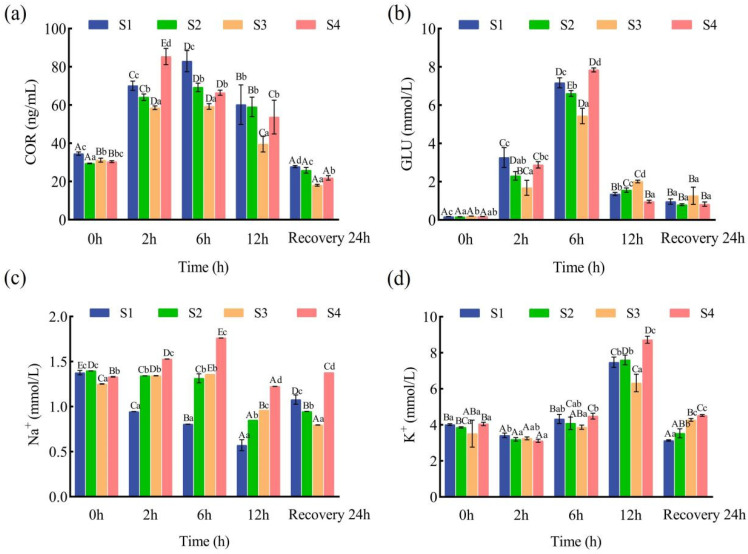
Effect of salt treatment on serum physiology and biochemistry for juvenile fish transportation. (**a**) serum cortisol (COR), (**b**) serum glucose (GLU), (**c**) serum Na^+^, (**d**) serum K^+^ levels. Data are presented as mean ± SD (*n* = 5); different capital letters represent significant differences between different sampling times under the same treatment conditions (*p* < 0.05), and different lower-case letters represent significant differences between different concentrations of pre-transportation salt bath treatments under the same sampling time (*p* < 0.05).

Variance analysis at various time points during transportation among the four salt bath treatment groups revealed distinct trends in serum biochemical indicators. In the S1 and S2 groups, juvenile serum COR levels increased significantly from 0 to 6 h of transport (*p* < 0.05), followed by a significant decrease from 6 to 12 h (*p* < 0.05). In the S4 group, COR levels peaked at 2 h (*p* < 0.05) and remained lower than those in the other groups thereafter. After 24 h of recovery, COR levels in all groups returned to baseline, with the S3 group exhibiting the lowest levels (*p* < 0.05). Between-group comparisons indicated that, at all time points during simulated transport, the S3 group had significantly lower COR levels than the other groups (*p* < 0.05; Figure 2a). Within-group analysis revealed that, in the S1 and S2 groups, juvenile serum GLU levels increased significantly from 0 to 6 h of transport (*p* < 0.05). In the S3 and S4 groups, GLU levels rose significantly from 0 to 6 h (*p* < 0.05) and decreased significantly from 6 to 12 h (*p* < 0.05). At 0 h, GLU levels in all groups were significantly lower than at subsequent time points (*p* < 0.05). After 24 h of recovery, GLU levels in all groups were significantly higher than at 0 h (*p* < 0.05; Figure 2b).

For serum Na^+^ concentrations, within-group comparisons showed a significant decrease in the S1 group with increasing transport time (*p* < 0.05). In the S3 and S4 groups, Na^+^ concentrations increased significantly from 0 to 6 h (*p* < 0.05) and decreased significantly from 6 to 12 h (*p* < 0.05; Figure 2c). Across all groups, serum K^+^ concentrations decreased from 0 to 2 h, increased from 2 to 6 h, and peaked at 12 h. After 24 h of post-transport recovery, serum potassium concentrations in all groups returned to pre-transport levels, whereas only the S4 group’s sodium concentration fully recovered. Between-group comparisons demonstrated that, at 12 h of simulated transport, the S3 group had the lowest serum K^+^ concentration among all treatment groups (*p* < 0.05; Figure 2d).

Overall, these findings demonstrate that pre-transport salt bath treatment, particularly in the S3 group (5‰ salt bath), significantly alleviated transport-induced elevations in serum cortisol and glucose and helped maintain sodium and potassium homeostasis. The persistent increase in stress markers and impaired ion regulation observed in the S1 group (0‰ salt bath) indicate greater physiological and metabolic disturbance. In contrast, the S3 group exhibited enhanced stress resilience and faster post-transport recovery, highlighting the protective effect of moderate salt pretreatment.

### 3.3. Liver Antioxidant Activity

**Figure 3:**
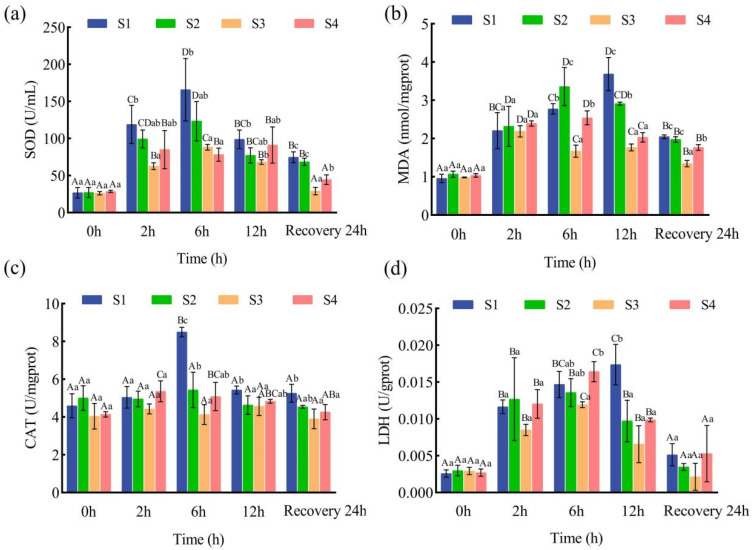
Effect of salt treatment on the antioxidant activity in the liver of juvenile fish transportation. (**a**) Superoxide dismutase enzyme activity in liver (SOD), (**b**) malondialdehyde content in liver (MDA), (**c**) catalase enzyme activity in liver (CAT), (**d**) lactate dehydrogenase enzyme activity in liver (LDH). Data are presented as mean ± SD (*n* = 5); different capital letters represent significant differences between different sampling times under the same treatment conditions (*p* < 0.05), and different lower-case letters represent significant differences between different concentrations of pre-transportation salt bath treatments under the same sampling time (*p* < 0.05).

Within-group analyses of juvenile liver SOD activity showed an initial increase, followed by a decline during transport. Among the salt bath treatments, the S3 group exhibited the lowest SOD activity after 2 h of simulated transport (*p* < 0.05), while S2 and S4 showed no significant difference throughout the period (*p* > 0.05). Notably, SOD activity in the S1 group remained consistently higher than in the other groups during both transport and recovery (Figure 3a).

For MDA content, no significant differences were observed among groups within the first 2 h (*p* > 0.05). However, after 6 h of transport and after 24 h of recovery, the S3 group had the lowest MDA levels among all treatments (*p* < 0.05; Figure 3b).

At the beginning of transport (0 h), CAT activity was at baseline across all groups, with no significant intergroup differences (*p* > 0.05). As transport progressed, CAT activity generally increased and then decreased. The S1 and S2 groups reached peak CAT levels at 6 h, with S1 showing the highest and S3 showing the lowest activity at this point. For all groups, except S1 and S4, CAT activity at 6 h was not significantly different from pre-transport levels (*p* > 0.05; Figure 3c).

LDH activity analysis revealed a continuous increase in the S1 group, peaking at 12 h (*p* < 0.05). Throughout the transport period, S1 maintained the highest LDH activity, while S2 and S4 showed no significant differences from each other (*p* > 0.05). S3 consistently demonstrated significantly lower LDH activity compared to the other groups (Figure 3d).

After 12 h of simulated transport and 24 h of recovery, only the S3 group showed no significant changes in CAT, SOD, or LDH activities compared to pre-transport levels (*p* > 0.05), indicating that a 5‰ salt bath (S3 group) effectively mitigated transport-induced stress responses.

### 3.4. IBR Indices

**Figure 4:**
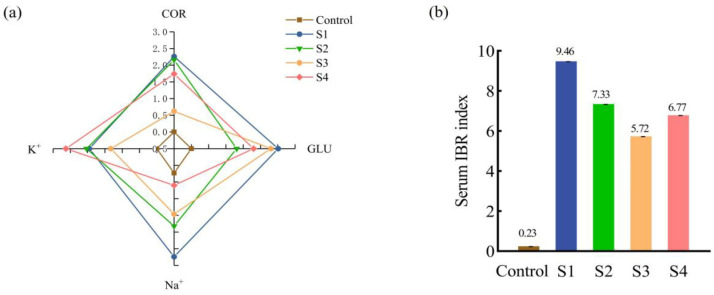
Effect of salt treatment on the serum IBR index of juvenile fish transportation. (**a**) IBR star chart, (**b**) IBR index chart.

After 12 h of simulated transport, the serum IBR data for juvenile Ictalurus punctatus under different salt bath treatments are shown in Figure 4. The four serum biomarkers (COR, GLU, Na^+^, K^+^) are plotted along separate axes of the radar chart, with the enclosed area representing the IBR value. All treatment groups (S1, S2, S3, and S4) displayed greater areas than the control group, with S1 showing the largest, followed by S4, S2, and S3 (Figure 4a). The serum IBR values of all salt bath groups were higher than those of the control (Control = 0.23), with S1 having the highest IBR (S1 = 9.46) and S3 having the lowest (S3 = 5.72). Notably, a larger IBR area—such as that observed in S1—reflects a heightened and more variable physiological stress response under transport conditions without salt pretreatment. Conversely, the smaller IBR area and lowest IBR value in S3 indicate a more stable physiological state and reduced systemic stress during transport. This suggests that a 5‰ salt bath prior to transport can effectively stabilize internal homeostasis and mitigate the overall physiological impact of transport stress (Figure 4b).

**Figure 5:**
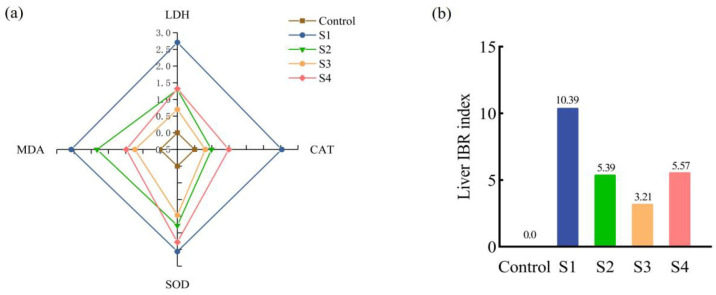
Effect of salt treatment on the liver IBR index of juvenile fish transportation. (**a**) IBR star chart, (**b**) IBR index chart.

As shown in Figure 5a, the four liver biomarkers (LDH, CAT, SOD, and MDA) were distributed along four axes in the radar chart, with the area representing the liver IBR value. The control group had the smallest area, while S1 had the largest. The biomarker areas in the livers of the S2, S3, and S4 groups were smaller than those in the S1 group. The liver IBR values of S2, S3, and S4 differed from those of S1, with S2 showing the greatest difference. Among the three salt bath groups, S2 and S4 had higher liver IBR values than S3 (Figure 5b). The higher liver IBR in S1 further indicates substantial hepatic oxidative stress and injury resulting from transport without salt pretreatment. In contrast, the notably smaller IBR area in the S3 group suggests improved hepatic antioxidant defense and minimized oxidative damage, reinforcing the protective effect of moderate (5‰) salt pretreatment at both systemic and organ levels.

### 3.5. Organological Structure

**Figure 6:**
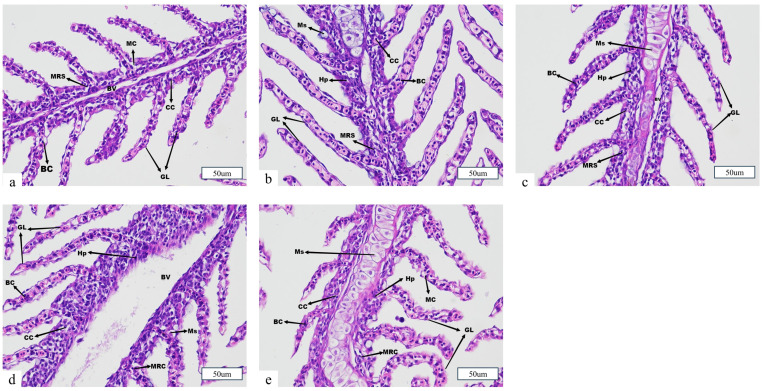
Effect of salt treatment on the gill morphology of juvenile fish transportation. (**a**) Control, (**b**) S1 (0‰), (**c**) S2 (1‰), (**d**) S3 (5‰), (**e**) S4 (9‰). Note: BC, blood cells; MRC, mitochondrial rich cell; GL, gill lamella; CC, chloride cells; BV, blood vessels; MC, mucus cell; Ms, swollen mucus cells; Hp, hyperplasia; Scale bars = 50 μm.

The changes in gill tissue structure of juvenile *Ictalurus punctatus* from the control and various salt bath concentration groups after 12 h of simulated transportation are shown in Figure 6. In the control group, the gill structure was intact, with well-organized and aligned gill filaments, abundant mitochondria, and normal chloride cells. After 12 h of simulated transportation, the S1 group (0‰ salt bath) exhibited swollen mucous cells, severe vacuolization and damage of chloride cells, pronounced cell proliferation in the gill filaments, and severe damage to red blood cells in the gill lamellae. These pathological changes indicate heightened mucosal stress and extensive gill injury under transport conditions without salt pretreatment. In the S2 group (1‰), cell proliferation in the gill filaments was also observed but was less severe than in the S1 group, suggesting a moderate protective effect at low salinity. In the S3 group (5‰), only mild damage to red blood cells in the gill lamellae and minimal cell proliferation were observed, indicating that moderate salt pretreatment effectively alleviated transport-induced gill injury and maintained better gill integrity. In the S4 group (9‰), the gill lamellae displayed various degrees of bending and irregular arrangement, accompanied by swollen mucous cells and markedly swollen red blood cells, reflecting that excessively high salt concentrations may also compromise gill health during transport.

**Figure 7:**
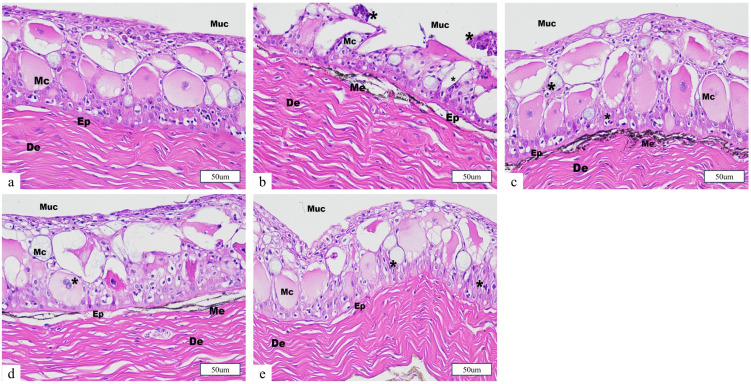
Effect of salt treatment on the skin morphology of juvenile fish transportation. (**a**) Control, (**b**) S1 (0‰), (**c**) S2 (1‰), (**d**) S3 (5‰), (**e**) S4 (9‰). Note: De, desmosomes; Ep, epidermis; Me, melanophore; Mc, mucus cells; Muc, mucous layer; *, hypertrophied mucous cells; Scale bars = 50 μm.

Histological analysis of the skin demonstrated that juvenile fish in the control group exhibited normal epidermal architecture, with intact epithelial and dermal layers. In contrast, all salt bath treatment groups displayed varying degrees of epidermal alteration after 12 h of transport. Notably, the S1 group (0‰ salt bath) showed pronounced mucous cell hyperplasia, epidermal erosion, epithelial cell detachment, and significant thinning of the epidermal layer. These pathological changes indicate that the absence of salt pretreatment leads to elevated cutaneous stress and compromised skin barrier function under transport conditions. The S2 (1‰) and S4 (9‰) groups also exhibited mucous cell hyperplasia and moderate epidermal disruption, suggesting that both low and high salinity provide only partial protection against transport-induced epithelial injury. In contrast, the S3 group (5‰) presented with the mildest histological alterations, displaying only mild mucous cell hyperplasia and largely preserved epidermal structure, implying that pretreatment with moderate salinity confers superior protection against transport-related skin damage in juvenile Ictalurus punctatus (Figure 7).

**Figure 8:**
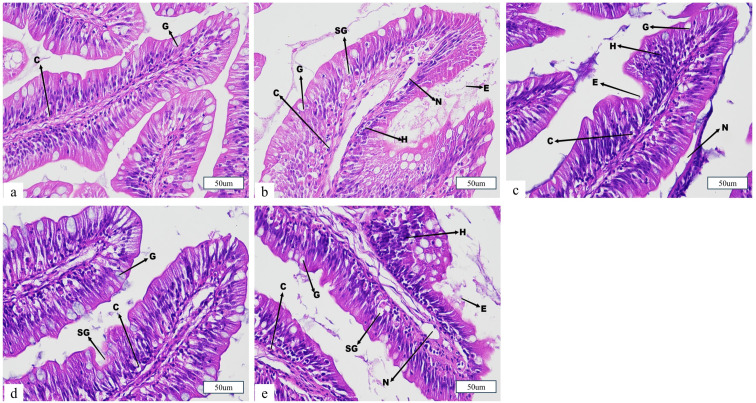
Effect of salt treatment on the morphology the gut of juvenile fish transportation. (**a**) Control, (**b**) S1 (0‰), (**c**) S2 (1‰), (**d**) S3 (5‰), (**e**) S4 (9‰). Note: C, columnar cells; G, goblet cells; SG, swelling of goblet cells; N, necrosis in the mucosal layer; E, erosion of villi; H, Hyperplasia; Scale bars = 50 μm.

Regarding the gut, juvenile fish in the control group exhibited normal intestinal architecture, with no observable pathological alterations in either columnar or goblet cells. After 12 h of simulated transportation, all salt bath treatment groups showed varying degrees of intestinal injury. In the S1, S2, and S4 groups, histological damage was characterized by goblet cell hypertrophy, increased cellular proliferation, swelling of the mucosal layer, and villous erosion. These alterations suggest heightened mucosal stress and impaired intestinal barrier function, particularly in the absence of salt pretreatment or at suboptimal salinity levels. In contrast, the S3 group (5‰) displayed only goblet cell hypertrophy, while the overall intestinal structure remained largely intact, indicating that moderate salt bath pretreatment effectively alleviates transport-induced intestinal damage and preserves mucosal integrity in juvenile Ictalurus punctatus (Figure 8).

## 4. Discussion

### 4.1. Effects of Salt Treatment on Water Quality and Survival Rates for Juvenile Fish Transportation

Water quality deterioration, including the accumulation of ammonia nitrogen, pH decline, and fluctuations in oxygen levels, represents a major challenge during live fish transport and is a key source of transport-induced stress [28]. Ammonia, released through fish metabolism, exists in both ionized (NH^4+^) and non-ionized (NH_3_) forms and is highly toxic due to its ability to readily penetrate tissues, disrupt oxygen transport, and impair physiological homeostasis [29,30]. Additionally, CO_2_ buildup from respiration leads to acidification and a drop in pH, further exacerbating stress and mortality risks [31]. In the present study, all juvenile *Ictalurus punctatus* survived both 12 h of simulated transport and 24 h of post-transport recovery, confirming that the tested salinities were within a safe range. Continuous monitoring revealed that, while TAN increased and pH declined over time in all groups, these changes were significantly attenuated in the salt bath treatment groups (S2, S3, S4) compared to the no-salt group (S1). Notably, after 12 h of simulated transport, the S3 group (5‰ salt bath) exhibited the lowest TAN and highest pH among all treatments (*p* < 0.05, *n* = 5 per group per time point). These results align with previous findings that salt addition reduces ammonia accumulation and helps maintain higher pH in the transport water of *Cyprinus carpio* and other species [32]. The pronounced benefits observed in the S3 group may be attributed to a 30 min pre-transport exposure to 5‰ salinity, which likely enhanced osmotic balance, reduced nitrogen excretion, and suppressed metabolic waste accumulation. This not only limited water turbidity and acidification but also minimized physiological stress, as reflected in the more stable water quality parameters compared to the S1 group. Conversely, the rapid rise in TAN and decline in pH in the no-salt group (S1) reflect greater metabolic stress and waste buildup, placing additional physiological burden on the fish during transport. In summary, these findings indicate that a pre-transport salt bath at the optimal concentration of 5‰ is an effective strategy for maintaining water quality, reducing the accumulation of toxic metabolites, and supporting the survival and welfare of juvenile channel catfish during live transport.

### 4.2. Effects of Salt Treatment on Serum Physiology and Biochemistry for Juvenile Fish Transportation

Upon exposure to environmental stressors, fish rapidly activate their internal defense mechanisms, with COR secretion representing one of the earliest hormonal responses. Variations in COR levels serve as an indirect indicator of the stress intensity experienced by fish [33]. Previous studies have established that transport stress elevates COR concentrations in fish [34]. In the present study, although COR levels in all groups ultimately returned to baseline following recovery, juveniles treated with a 5‰ salt bath (S3) exhibited significantly lower COR levels during transport (*p* < 0.05). This reduction is likely due to the salt bath’s capacity to alleviate or prevent osmotic disturbances arising from pre-transport handling, thereby mitigating the overall stress response. These findings are consistent with previous observations in Nile tilapia (*Oreochromis niloticus*) [35].

Under stress, GLU concentrations in fish rise markedly, making GLU a key indicator of stress responses [36]. During transport, GLU levels first increased and then declined, a trend consistent with Wang et al. [37] for *Megalobrama amblycephala* under transport stress. The stress-induced elevation in GLU is linked to increased catecholamine release, which acts on the liver to promote glycogenolysis, gluconeogenesis, and glycolysis, ultimately raising blood GLU levels [38]. As transport progresses, a decline in GLU may reflect deteriorating water quality, which accelerates metabolism and depletes energy reserves [39]. Notably, the S1 group (0‰, no salt) showed the most pronounced and sustained GLU elevation, suggesting persistent physiological disruption and impaired recovery capacity. All groups exhibited initial increases in COR and GLU during simulated transport, indicative of hypothalamic–pituitary–interrenal (HPI) axis activation and energy mobilization [40]. However, the S3 group’s peak COR and GLU levels remained consistently lower than those in either the low-salinity (S2, 1‰) or high-salinity (S4, 9‰) groups, with more rapid normalization, indicating a superior ability to suppress acute stress and facilitate metabolic recovery. These results agree with previous research demonstrating that moderate salt supplementation stabilizes the stress response in other aquaculture species by minimizing osmotic and handling disturbances [35,36,37].

Alterations in serum Na^+^ and K^+^ concentrations during simulated transport reflect the impact of stress on osmoregulatory function. Transport stress can rapidly induce catecholamine release, alter serum ion diffusion, and increase gill permeability, leading to greater water influx and ion loss [32]. In this study, S1 fish exhibited a significant decrease in serum Na^+^ (*p* < 0.05) and a significant increase in K^+^ throughout transport, consistent with the findings of Biswal et al. [41] for juvenile *Labeo rohita* and indicating a breakdown in osmoregulation. In the S3 and S4 groups, Na^+^ levels initially increased and then decreased, while K^+^ levels steadily rose, suggesting a delayed effect of the salt bath on osmotic disturbances. The observed patterns for Na^+^ and K^+^ demonstrate the dose-dependent benefits of the salt bath: the S3 group (5‰) maintained the most stable ion levels—both Na^+^ and K^+^ remained within physiological ranges throughout transport and recovery—outperforming both the S2 (1‰) and S4 (9‰) groups. Excessive salinity (S4, 9‰) may disrupt ionic gradients, offering no additional benefit over S3 and occasionally delaying Na^+^ recovery, suggesting that overly high salinity can introduce extra ionic stress instead of protection.

This study demonstrates that a 5‰ salt bath (S3) most effectively alleviates transport-induced physiological stress in juvenile Ictalurus punctatus by stabilizing key serum biomarkers (COR, GLU) and maintaining ionic homeostasis (Na^+^, K^+^). The S3 protocol provides an optimal balance—substantially reducing both endocrine and ionic disruptions associated with live fish transport while avoiding the adverse effects of insufficient (S1) or excessive (S4) salt exposure. These results highlight the value of adopting a 5‰ salt bath protocol in aquaculture to maximize fish health and stress resistance during transport.

### 4.3. Effects of Salt Treatment on the Antioxidant Activity in the Liver of Juvenile Fish Transportation

Reactive oxygen species (ROS) are byproducts of normal metabolism in animals and play essential roles in intracellular signal transduction and apoptosis [42]. However, excessive ROS accumulation induces oxidative stress, leading to damage of cellular proteins, lipids, and DNA, which can ultimately result in organismal death [43]. SOD catalyzes the conversion of excess ROS into hydrogen peroxide (H_2_O_2_), while CAT further converts H_2_O_2_ into H_2_O and O_2_ for physiological utilization [44]. The findings of this study indicate that, both during simulated transport and after 12 h of transport, CAT and SOD activities in the S2, S3, and S4 groups were generally lower than those in the no-salt control group (S1), suggesting that pre-transport salt baths can decrease ROS production and thus mitigate oxidative stress during transport. This protective effect was most pronounced in the S3 group, which consistently showed the lowest SOD and CAT activities, implying that moderate salt pretreatment provides a more stable redox environment and reduces oxidative damage. Compared to lower (S2, 1‰) and higher (S4, 9‰) salt concentrations, the 5‰ salt bath (S3) was more effective in preventing excessive upregulation of antioxidant enzymes, thereby supporting optimal redox balance under transport stress. MDA, a final product of lipid peroxidation, is commonly used as an indicator of oxidative stress-induced damage. In this study, MDA levels in the S3 group were significantly lower than those in the control group (S1) after 6 h of transport (*p* < 0.05), further supporting the dose-dependent protective effect of a moderate salt bath against free radical damage, with 5‰ salt providing the best suppression of oxidative stress. LDH is a key biomarker of anaerobic energy metabolism in fish [39]. After 12 h of transport, the S1 group showed significantly higher LDH activity than the S2, S3, and S4 groups (*p* < 0.05), indicating that deteriorating water quality and prolonged transport stress increased energy demands for metabolic homeostasis in juveniles [45]. At all time points, the S3 group maintained the lowest and most stable LDH activity, outperforming both S2 and S4 in sustaining energy metabolic homeostasis during transport and recovery. Collectively, these results demonstrate that, among all salt bath treatments, the 5‰ salt bath (S3) was most effective at maintaining redox and metabolic homeostasis in juvenile *Ictalurus punctatus*, as evidenced by the lowest and most stable values of SOD, CAT, MDA, and LDH throughout the simulated transport process. The superior performance of S3 compared to the low- and high-salinity groups suggests that moderate salt pretreatment confers optimal protection against transport-induced oxidative and metabolic stress, providing practical recommendations for the application of salt baths in aquaculture transport protocols.

### 4.4. Effects of Salt Treatment on the IBR Index of Juvenile Fish Transportation

When fish encounter transport-related environmental stressors [46], they activate a complex array of defense mechanisms to maintain internal homeostasis [47]. However, assessing only a single hormone or enzyme often fails to fully capture the organism’s comprehensive stress response [48]. The integrated biomarker response (IBR) index, which aggregates data from multiple enzymes, hormones, and other biomarkers, enables a more objective and quantitative evaluation of stress levels in fish [49,50]. Recently, the IBR has emerged as a valuable tool for optimizing the dosage of sodium chloride used as a water additive in aquaculture transport systems [42]. In this study, eight biomarkers—COR, GLU, Na^+^, K^+^, LDH, CAT, SOD, and MDA—were analyzed in the serum and liver tissues of juvenile *Ictalurus punctatus* after 12 h of simulated transportation using the IBR model. All salt bath treatment groups (S2, S3, S4) exhibited lower IBR indices than the no-salt control group (S1), with the S3 group (5‰) consistently showing the smallest IBR area and value. The larger IBR area observed in S1 indicates heightened and more variable physiological stress and greater organ damage in the absence of salt pretreatment. Conversely, the markedly smaller IBR area in S3 reflects a more stable physiological state, reduced systemic stress, and enhanced hepatic antioxidant defenses during and after transport. Collectively, these findings indicate that a moderate (5‰) salt bath confers the most significant protective effects at both systemic and organ levels by strengthening antioxidant defenses and reducing oxidative damage. From a practical perspective, a 30 min, 5‰ salt bath prior to transport is an effective, low-cost, and operationally simple strategy to enhance fish welfare and survival during commercial transportation.

### 4.5. Effects of Salt Treatment on the Organizational Morphology of Juvenile Fish Transportation

The gills are the primary respiratory organs in fish, responsible for gas exchange. Additionally, they play vital roles in maintaining osmotic balance, eliminating ammonia nitrogen waste, and regulating body pH [50]. The integrity of gill tissue morphology is essential for normal physiological function in fish [51]. In this study, simulated transportation caused varying degrees of damage to the gill tissue structure of juvenile *Ictalurus punctatus*, including mucous cell swelling and hyperplasia, bending and breakage of gill filaments, and rupture of red blood cells. Among all transport groups, the S1 and S4 groups exhibited severe gill tissue damage, while the S2 and S3 groups showed milder damage. The skin plays a crucial role in maintaining internal environmental balance and protecting against pathogen invasion [50]. The results of this study revealed that, after 12 h of simulated transportation, the S1 group exhibited the most significant skin tissue damage, while the S2, S3, and S4 groups exhibited varying degrees of damage, with the S3 group showing the least damage. This could be attributed to the pre-transport salt bath, which may have enhanced the secretion of mucus on the skin surface of juvenile fish, thereby alleviating environmental stressors, such as crowding and turbulence, during transportation and promoting gas and ion exchange [52]. The intestine plays a critical role in nutrient absorption and digestion and serves as the first line of defense against pathogen invasion, which is essential for fish health [53,54,55]. Thus, the integrity of the intestinal structure is vital for its proper function. Histological results indicated that, under transportation stress, the S1 group suffered more severe damage to the intestinal structure, consistent with the findings reported by Wang et al. in their study on *Micropterus salmoides* [56]. Although the S4 group showed improved serum ion regulation, histological evidence indicated potential tissue irritation or osmotic overload at this concentration. In conclusion, this study found that transportation caused significant damage to the gill, skin, and intestinal structures, with the severity of damage varying depending on the pre-transport salt bath concentration. Optimizing transportation conditions can help maintain the integrity of *Ictalurus punctatus* juveniles’ tissues and mitigate transport-induced stress. Among the current concentration gradients, the 5‰ pre-transport salt bath (S3 group) was the most effective in alleviating transport stress.

## 5. Conclusions

This study demonstrates that a 30 min pre-transport salt bath, especially at a concentration of 5‰ (S3), offers substantial protection to juvenile *Ictalurus punctatus* during live fish transport. Fish receiving salt bath treatment showed enhanced water quality—evidenced by lower total ammonia nitrogen and higher pH—along with reduced cortisol levels and decreased histological damage to gill, skin, and intestinal tissues relative to untreated controls. Analysis using the integrated biomarker response (IBR), which provides a comprehensive assessment of physiological and oxidative stress, further verified that the 5‰ salt bath group maintained the most stable physiological state and experienced the lowest cumulative stress. Collectively, these findings indicate that a 5‰ pre-transport salt bath significantly improves the stress resilience and welfare of juvenile *Ictalurus punctatus* during transport, with minimal cost and operational complexity. The results support the practical application of moderate salt bath protocols in aquaculture to enhance transport success and fish survival.

## Data Availability

Data will be made available on request.

## Supplementary Materials

The following supporting information can be downloaded at: https://www.mdpi.com/article/10.3390/ani15152249/s1, Figure S1: Probability of Survival and post-transport cortisol levels after treatment in different salt bath concentrations for different durations. Probability of Survival (a) and cortisol (COR) levels (b).
