# Supplementary material for: Pre-Transport Salt Baths Mitigate Physiological Stress and Tissue Damage in Channel Catfish (Ictalurus punctatus) Fingerlings: Evidence from Multi-Biomarker Assessment and Histopathology
Source: Animals (Basel). 2025 Jul 31;15(15):2249. doi: 10.3390/ani15152249 (PMC12345568; doi:10.3390/ani15152249)
Supplement: Supplementary file 1 [file animals-15-02249-s001.zip › animals-3739307-supplementary.pdf]

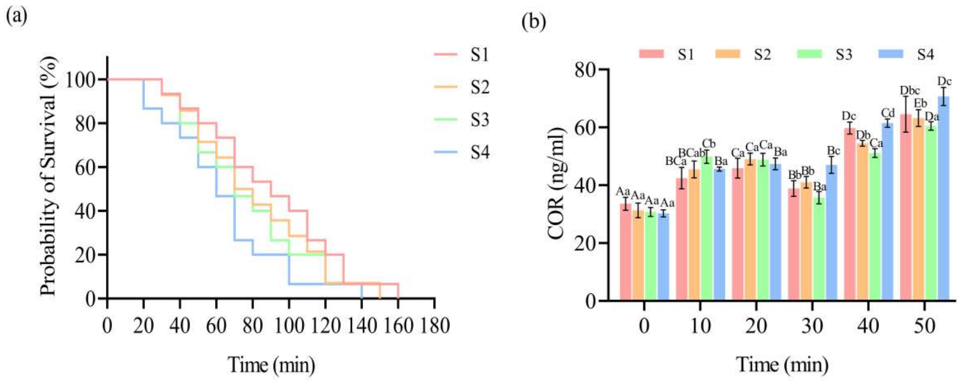

Figure S1. Probability of Survival and post-transport cortisol levels after treatment in different salt bath concentrations for different durations

Probability of Survival (a) and cortisol (COR) levels (b)

Data are presented as mean ± standard deviation. Different capital letters indicate significant differences among different salt bath times under the same treatment conditions ($P < 0.05$), and different lowercase letters indicate significant differences among different salt bath concentrations under the same salt bath time ($P < 0.05$).
